# Impact of Vitamin B1 and Vitamin B2 Supplementation on Anxiety, Stress, and Sleep Quality: A Randomized, Double-Blind, Placebo-Controlled Trial

**DOI:** 10.3390/nu17111821

**Published:** 2025-05-27

**Authors:** Yingxuan Tao, Murong Wu, Boyao Su, Heng Lin, Qianzi Li, Tian Zhong, Ying Xiao, Xi Yu

**Affiliations:** Faculty of Medicine, Macau University of Science and Technology, Avenida Wai Long Taipa, Macau; 2230017837@student.must.edu.mo (Y.T.); 2240002221@student.must.edu.mo (M.W.); 2230017878@student.must.edu.mo (B.S.); 2230004854@student.must.edu.mo (H.L.); 2230027731@student.must.edu.mo (Q.L.); tzhong@must.edu.mo (T.Z.); yxiao@must.edu.mo (Y.X.)

**Keywords:** mental health, anxiety, stress, sleep quality, vitamin B1, vitamin B2

## Abstract

*Background*: Anxiety, stress, and sleep disturbances significantly affect overall health. Research suggests that vitamins B1 and B2 may play a role in mood regulation and neuroprotection. This study aimed to investigate the effects of vitamin B1 and B2 supplementation in alleviating anxiety and stress and improving sleep quality. *Methods*: This study was a parallel randomized, double-blind, placebo-controlled clinical trial. Participants (n = 43) were randomized to receive one of the following two interventions: 100 mg of vitamin B1 and 100 mg of vitamin B2 or placebo. Intervention outcomes were assessed at baseline and week four, including SAS (Self-Rating Anxiety Scale), PSS (Perceived Stress Scale), PSQI (Sleep Quality Index), ESS (Sleepiness Scale), and measurement of urinary vitamin B1 and B2 levels. *Results*: After four weeks, urinary vitamin B1 levels increased from 158 ± 108.9 ng to 1333.1 ± 1204.5 ng (*p* < 0.01), and urinary vitamin B2 levels increased from 308.0 ± 198.3 ng to 6123.2 ± 4847.2 ng in the supplement group (*p* < 0.01). The PSS scores decreased significantly in the supplement group from 21.5 ± 4.1 to 15.5 ± 4.5 (*p* < 0.05), while the placebo group showed a change from 20.3 ± 4.3 to 19.8 ± 5.5. Vitamins B1 and B2 did not have a significant effect on anxiety improvement (*p* > 0.05). The PSQI scores decreased in the supplement group from 8.0 ± 3.12 to 6.3 ± 2.0 (*p* < 0.05), while the placebo group worsened from 5.7 ± 2.7 to 7.4 ± 2.9. Meanwhile, the ESS scores in the supplement group decreased from 13.0 ± 3.4 to 9.1 ± 3.9 (*p* < 0.05), demonstrating a significant improvement compared to the placebo group. *Conclusions*: The clinical trial findings demonstrated that while vitamin B1 and B2 supplements helped reduce stress, enhance sleep, and reduce sleepiness, they had no discernible impact on reducing anxiety. Future studies should focus on the long-term effects of vitamin B1 and B2 supplements, exploring the combined effects of combined vitamin B1 and B2 medications for the treatment of stress and sleep disorders.

## 1. Introduction

Anxiety is one of the most prevalent mental illnesses in modern society [1]. In 2019, there were about 301 million people worldwide who suffered from anxiety, which accounted for about 4% of the world’s population. Another of the most prevalent mental health issues facing people today is stress, which has been called the “disease of the century” [2]. And higher levels of stress are linked to a higher risk of anxiety, even though stress is not a mental illness itself [3]. Anxiety and stress are psychological conditions that affect society, family, public health, and the quality of life.

The mechanisms of anxiety and stress are unclear. The relationship between anxiety and stress seems intuitive, but the definitions and neurophysiological processes of these two states are extremely different [4]. Anxiety is defined as a time-dispersed emotional state caused by potentially harmful situations, where the likelihood or frequency of harm is low or uncertain [5,6,7]. Historically, psychologists and psychiatrists have categorized anxiety into state anxiety and trait anxiety [5,6,7]. State anxiety is a heightened alertness to potential threats that may be triggered by acute stress, with its primary function being to avoid dangerous situations and promote memory consolidation [6,8]. Trait anxiety, on the other hand, is an individual’s tendency to express anxiety in a persistent manner, characterized by chronicity [6,8]. Stress is defined as the pressure response generated by real or perceived threats (stressors). It can be defined as an emergency state in which an organism responds to a challenge to its internal balance [9,10]. In this emergency state, the organism initiates a comprehensive response involving physiological and behavioral reactions [4]. Stressors can be categorized into two types: systemic stressors, also known as internal threats, which include physical changes in the body; and psychological stressors, also known as perceived threats, which include situations that may lead to danger and trigger challenges to homeostasis [11,12].

Although anxiety and stress have different definitions, anxiety and stress have a strong bidirectional relationship in terms of behavior and neurological basis. These commonalities are crucial to understanding each state and its interactions [4]. Research revealed that numerous neurotransmitters, including serotonin, γ-aminobutyric acid (GABA), norepinephrine, and stress and anxiety, are thought to interact through certain mechanisms [13,14]. Particularly, as the principal inhibitory neurotransmitter, GABA has been studied for its interactions with anxiety [15], stress [14], and sleep [16] in the central nervous system. Other researchers have suggested that high levels of anxiety and stress trigger a disturbance in redox regulation in the body, promoting the secretion of stress hormones such as cortisol and adrenaline, leading to the production of excessive reactive oxygen species (ROS) by dysfunctional mitochondria, which impairs the body’s antioxidant mechanisms [17]. Overproduction of ROS also damages proteins, DNA, and cell membranes and speeds up the start of lipid peroxidation. Malondialdehyde, 4-hydroxynonenal (4-HNE), and other lipid peroxidation products can cause inflammation [18]. This exacerbates oxidative stress-induced brain damage by generating a vicious loop of ROS, reactive nitrogen species (RNS), and mitochondrial dysfunction [17]. Furthermore, some researchers suggested that the development of stress and anxiety may be linked to micronutrient shortages [19].

Stress and anxiety significantly impair the quality of sleep, which is shown by the change in components of sleep health, including duration, quality, regularity, and sleep disorders [20]. The prolonged hyperactivity of the brain brought on by anxiety makes it difficult to fall asleep. Stress causes the body to produce stress hormones and trigger a stress response, which impairs brain function and lowers the proportion of deep sleep, resulting in sleep deprivation [21]. In addition to raising the risk of immune system malfunction, metabolic disease, intestinal flora abnormalities, and cardiovascular disease, poor sleep quality is also linked to the development of disease by disrupting and intensifying stress and anxiety levels [22].

Apart from the side effects and high cost, the available treatment options for relieving anxiety and stress and improving sleep quality are not entirely effective. Because of their safe and natural qualities, alternative medicines like vitamins, minerals, and nutritional supplements have become popular [23]. Vitamin B1 and vitamin B2 are both necessary micronutrients. Through their capacity to fight oxidative stress, enhance neurotransmitter synthesis, and promote cellular energy metabolism, vitamins B1 and B2 protect brain areas linked to mood and sleep disorders [24]. This implies that mood swings and sleepiness may be related to vitamin B1 and vitamin B2.

Vitamin B1 and vitamin B2 supplementation can help reduce anxiety and stress and improve sleep quality. Early research mainly focused on single-vitamin supplementation; a placebo, double-blind, randomized controlled trial demonstrated that vitamin B1 supplementation alone effectively reduced depression and anxiety in patients with major depressive disorder [25]. More recently, research has looked at the combined effects of combining multiple nutrients; a cross-sectional study found that dietary intake of B vitamins in a population was linked to a lower prevalence of anxiety and stress [26], and a multicenter randomized clinical trial found that vitamin B2 and succinic acid showed great efficacy in reducing anxiety and stress by increasing antioxidant enzyme activity to achieve relief from diabetic peripheral neuropathy (DPN) problems [27]. Based on an interventional trial, taking supplements of vitamins B1 and B6 combined was linked to improved sleep quality, reduced anxiety, and cognitive impairments [28]. Similarly, another clinical study found that giving night shift workers a multivitamin and vitamin B complex supplements enhanced their quality of sleep [29]. The potential for biological synergy between B vitamins combined intervention is highlighted by this gradual transition from single-vitamin methods to multi-nutrient combinations (e.g., vitamin B1 and vitamin B2).

Studies provide stronger population-based evidence supporting the effects of vitamin B1 and B2 supplements, particularly when used in combination, suggesting interactions with anxiety, stress, and sleep quality. To the best of our knowledge, most population-based studies have primarily focused on specific categories of vitamins or common vitamins, such as vitamin C, vitamin D, and vitamin B6 and B12 [30,31,32]. Research on vitamin B1 is limited, and most studies involving vitamin B1 have been general assessments conducted within the broader B-complex vitamin group [33,34]. In addition, research on vitamin B2 has mainly focused on its clinical trials for alleviating primary neurological disorders, especially migraines [35,36]. However, no studies have reported the combined use of vitamins B1 and B2. Both vitamins belong to the B-complex family, and their combined effects warrant further exploration. This research challenge highlights the novelty of this study, which seeks to explore the combined effects of vitamin B1 and B2 supplementation to investigate their interactions and combined mechanisms in improving mental health and sleep quality, an area that has not been systematically studied previously.

Therefore, considering the limitations and inconsistencies of data from previous studies, the presented study was conducted to explore the effects of vitamin B1 and vitamin B2 combined supplementation on reducing anxiety and stress and increasing individual sleep quality. This study was conducted as a randomized, controlled trial to provide stronger evidence of causal inference, supported by population data, and to provide a stronger scientific foundation.

## 2. Materials and Methods

### 2.1. Study Design and Setting

This study was a parallel randomized, double-blind, placebo-controlled clinical trial designed to assess the effects of a nutritional intervention of vitamin B1 and vitamin B2 versus placebo on the alleviation of anxiety and stress and the enhancement of sleep quality in a college population. The study was carried out from January 2025 to March 2025 at Macau University of Science and Technology, Macau.

### 2.2. Participants

Volunteers aged 18–25 years old were recruited from the university, Macau, through campus advertisements or social media. Interested participants were first screened through on-site screening and were required to meet the following inclusion criteria: (1) aged between 18 and 25 years old; (2) Self-Rating Anxiety Scale (SAS) score > 44; (3) Perceived Stress Scale (PSS) score > 13; (4) Pittsburgh Sleep Quality Index (PSQI) score > 5; (5) Epworth Sleepiness Scale (ESS) score ≥ 6. Excluded from this study were (1) individuals with a BMI below 18.5 and over 40 kg/m^2^; (2) individuals with urinary vitamin B1 levels greater than 200 µg and urinary vitamin B2 levels greater than 800 µg; (3) individuals experiencing stressors such as bankruptcy, failure in love, and a history of death of a first-degree relative and close friend within the past six months; (4) individuals suffering from neurological or organic disorders; (5) individuals suffering from pathological anxiety (e.g., generalized anxiety disorder GAD) or psychiatric disorders (e.g., post-traumatic stress disorder); (6) individuals diagnosed with inflammatory disorders and other specific diseases such as diabetes mellitus, cardiovascular, cancer, hypertension, renal disorders, liver disease, hyperthyroidism, epilepsy, and multiple sclerosis (MS); (7) individuals diagnosed with pathological sleep disorders (e.g., severe insomnia, severe obstructive apnea sleep apnea syndrome); (8) recent history of alcohol and drug use (at least within past 3 months); (9) pregnant or lactating women; and (10) allergy to vitamin B1 and vitamin B2 tablets. In addition, subjects who failed to complete the questionnaire and follow the guidelines correctly were excluded.

To find subjects who met the criteria, 84 volunteers were first introduced to the study, and 10 withdrew (4 refused to participate and 6 could not be contacted). Seventy-four were invited to assess anxiety and stress status via the SAS and PSS; PSQI and ESS, respectively, of which 16 dropped out (2 did not submit the scales at the designated time, 4 with highly repetitive options were deemed invalid scales, and 10 with SAS < 50, PSS < 13, PSQI < 6, and ESS < 6 were identified as meeting the exclusion criteria). Fifty-eight individuals were then invited to confirm vitamin B1 and vitamin B2 in vivo nutritional levels. Researchers collected all urine samples from participants’ 4-h vitamin urine loading tests and performed laboratory measurements, and 8 people dropped out (>200 µg of vitamin B1 in urine and >800 µg of vitamin B2 in urine). Finally, 50 people met the inclusion and exclusion criteria to be selected as participants and included in the study.

Considering α = 0.05 and β = 0.20 (power = 80%) to detect small to moderate effect size = 0.36 based on previous studies [28], the estimated minimum sample size in each group was 20. To cover up the possible withdrawal, 22 participants for each group were considered.

### 2.3. Randomization and Blinding

Using computer software, random numbers were used to generate a random assignment cohort, and subjects were randomly assigned to either the vitamin B1 and vitamin B2 nutritional supplementation group or placebo in a 1:1 ratio. Randomization and coding of the nutritional supplement and placebo groups were performed by a third person. Therefore, all participants and researchers were unaware of the allocation until the end of the study.

### 2.4. Intervention

The study was divided into a vitamin B1 and a vitamin B2 nutritional supplement group and a placebo group. Volunteers in the nutritional supplement group received one vitamin B1 supplement tablet (100 mg/tablet) and one vitamin B2 supplement tablet (100 mg/tablet). The placebo group received two sucrose tablets with the same appearance, odor, color, taste, and packaging as the vitamin B1 and vitamin B2 supplement tablets. Participants were asked to take it daily with breakfast and continue for four weeks. The compliant vitamin B1 and vitamin B2 supplement tablets were manufactured by Now Foods, Inc. (Bloomingdale, IL, United States) under Good Manufacturing Practice (GMP) conditions. Now Foods is SSCI-certified by Intertek, indicating compliance with 21 CFR Part 111 and high standards for supplement safety and manufacturing consistency. The doses used in this study were based on doses used in similar studies in previous trials and were within the nutritional supplement safety range [28,37,38,39]. To ensure participant adherence, researchers will follow up with participants with daily phone calls, social software, and retrieve empty bags after one week. Subjects were asked to maintain a normal level of diet for 4 weeks. After intervention acceptance, participants were asked to complete the SAS, PSS, PSQI, and ESS scales alone and were interviewed by a professional researcher.

### 2.5. Outcome Measures

The primary outcome measures of this study were changes in anxiety, perceived stress, sleep quality, and sleepiness, assessed using the Self-Rating Anxiety Scale (SAS), the Perceived Stress Scale (PSS), the Pittsburgh Sleep Quality Index (PSQI), and the Epworth Sleepiness Scale (ESS), respectively. These measures were used to evaluate the effects of vitamin B1 and B2 supplementation on participants’ emotional status and sleep-related conditions. The secondary outcome measures were changes in urinary concentrations of vitamin B1 and B2, used to assess the biological responses to the intervention and changes in nutritional status.

### 2.6. Assessment of Vitamin B1 and Vitamin B2 Levels

Vitamin B1 and vitamin B2 nutrient reserve levels were assessed before and after the intervention by means of a vitamin urinary loading test. The 4-h urinary loading test was conducted in the Faculty of Medicine of the Macau University of Science and Technology (MUST). Participants were given one tablet of vitamin B1 (5 mg) and one tablet of vitamin B2 (5 mg) orally, drank 400–500 mL of water during the waiting period, and all urine was collected over a 4-h period to record the urine volume. Urine samples were immediately collected for laboratory testing, and the retained samples were labeled and stored in a 4-degree refrigerator. Vitamin B1 and vitamin B2 in urine were measured using the urine load test [40,41]. Due to the fluorescent properties of vitamin B1 and vitamin B2, their fluorescence signals can be detected by an enzyme labeling instrument (MEGAMAN^®^ (Shanghai) Co., Ltd. (Shanghai, China), SpectraMax iD3 Multi-Mode). Vitamin B1 can be measured at the excitation wavelength of 365 nm and emission wavelength of 435 nm; vitamin B2 can be measured at the excitation wavelength of 420 nm and emission wavelength of 530 nm for the fluorescence signal [42]. The amount of vitamin B1 or vitamin B2 in the urine sample is calculated from a pre-drawn standard curve.

### 2.7. Scale

(1) Self-Rating Anxiety Scale (SAS)

The Self-Rating Anxiety Scale (SAS), developed by American psychologist Zung in 1971 [43], is a self-report scale used to assess an individual’s feelings of anxiety. The SAS scale consists of a total of 20 items, which assess an individual’s symptoms of anxiety over the past week. The items in the scale cover somatic and emotional responses to anxiety, including nervousness, fear, rapid heartbeat, difficulty sleeping, and lethargy. Each entry consists of a rating of 1 (no or very little time), 2 (a small amount of time), 3 (quite a bit of time), and 4 (most or all of the time). Participants answered questions based on their feelings of anxiety. A raw total score was first calculated for each scale, and the raw score was multiplied by 1.25 to obtain a standardized score, which was a number between 25 and 100 [43].

(2) Perceived Stress Scale (PSS)

Developed by Cohen et al. in 1983 [44], the Perceived Stress Scale was designed to measure an individual’s subjective experience and perception of life stress over the past month and is a psychometric tool used to assess an individual’s perceived level of stress, which is widely used in clinical assessment and mental health research. The items in the scale cover an individual’s perception of stress in different situations in daily life, including the sense of control over life events, the frequency of stress, and the ability to cope with life events. Each item includes ratings of 0 (never), 1 (rarely), 2 (sometimes), 3 (often), and 4 (always). Participants answered these questions based on their own sense of stress, and a total score, a number between 0 and 40, was calculated for each scale [44].

(3) Pittsburgh Sleep Quality Index (PSQI)

The Pittsburgh Sleep Quality Index (PSQI) was developed in 1989 by Buysse et al. [45] at the University of Pittsburgh, USA, as a self-report scale for assessing the quality of an individual’s sleep. The PSQI has 19 entries with 7 components, including subjective sleep quality, sleep latency, sleep duration, sleep efficiency, sleep disorders, use of sleep medication, and daytime dysfunction. The rating range for each component included 0 (none), 1 (<1 time/week), 2 (1–2 times/week), and 3 (≥3 times/week). Participants answered these questions based on their sleep reality, and a scale score was calculated as a number between 0 and 21. Higher scores indicate poorer sleep quality [45].

(4) Epworth Sleepiness Scale (ESS)

Developed by Johns in Australia in 1991 [46], the Epworth Sleepiness Scale (ESS) is a self-report scale used to measure an individual’s daytime sleepiness. Designed to help identify possible sleep disorders, it can be used as a supplement to the daytime dysfunction component of the PSQI. The ESS consists of eight scenarios, each of which represents a daytime scenario that could lead to drowsiness. Each scenario is rated on a scale ranging from 0 (will not doze off), 1 (little likelihood of dozing off), 2 (moderate likelihood of dozing off), and 3 (very likely to doze off). Participants answered these questions based on their actual daytime sleepiness, and a scale score was calculated [46].

### 2.8. Statistical Analysis

All data analyses were performed using R Software (version 4.3.2; R Core Team, Vienna, Austria, 2023). We used mean ± standard deviation (SD) for quantitative variables and frequency (percentage) for qualitative variables. Within-group comparisons were analyzed using paired-samples *t*-tests. Between-group differences at baseline were assessed using independent samples *t*-tests. Between-group comparisons at post-intervention were conducted using ANCOVA, with baseline values and potential confounding variables included as covariates. Statistical significance was defined as *p* < 0.05.

### 2.9. Ethical Considerations

The study was approved by the Medical Ethics Committee of the Macau University of Science and Technology, Macau, under the ethical guideline MUST-FDCT-20240226001. The trial was registered with the China Clinical Trial Registry under the registration code ChiCTR2500096497: study participants were completely voluntary, and any participant had the option to withdraw from the study at any stage of the study. All study participants provided written informed consent. Subjects were not required to pay any fees for their participation.

## 3. Results

### 3.1. Participant Characteristics

**Figure 1:**
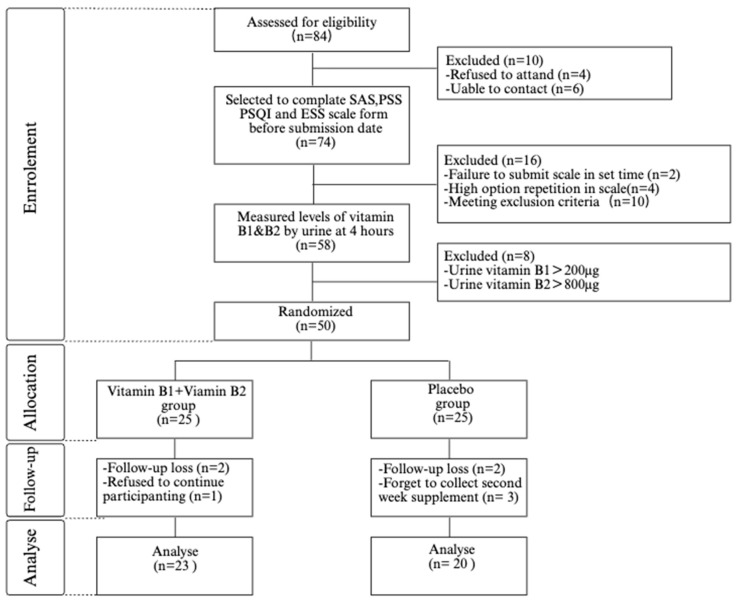
CONSORT flow diagram of participant enrollment, allocation, and analysis.

The participant recruitment process was as follows (Figure 1): A total of 50 participants were included in the study for randomization, with 25 participants in each of the vitamin B1 and B2 supplementation group and the placebo group according to the principle of randomization. Two participants in the intervention group did not return for follow-up for unknown reasons, and one participant withdrew consent without giving a reason. Two participants in the control group did not return for follow-up on time, and three participants were excluded for failure to comply with the study. Of the eight participants who withdrew, three and four participants completed the first and second week of assessments, respectively (all outcome indicators were available to them up to these time points). A total of 43 participants completed the 4-week study (23 in the supplementation group and 20 in the placebo group). During the study period, it was learned through daily online follow-up that participants did not report any side effects after supplementation with vitamins B1 and B2 and a placebo. No serious adverse events occurred. In the participant characteristics (Table 1), we observed that the number of participants in the vitamin B1 and B2 supplementation group and the placebo group were similar in terms of the number of participants, age, BMI, and education, and that the participants’ birthplaces were mostly from the southern region of China (78.2% versus 90%), and their dietary habits were more similar. In addition, baseline levels of vitamin B1 and vitamin B2 were relatively similar in the supplementation and placebo groups.

### 3.2. Vitamin B1 and Vitamin B2 Levels

According to the mean (SD) values of vitamin B1 and B2 levels presented in Table 2 and Table 3, participants in the intervention group had significantly higher levels of both vitamins at week 4 compared to the placebo group, although baseline levels were comparable between groups. After adjusting for potential confounding variables including age, BMI, and smoking status, using ANCOVA, the between-group differences in post-intervention levels remained statistically significant for both vitamin B1 (adjusted *p* = 0.039) and vitamin B2 (adjusted *p* = 0.004), indicating optimistic supplement effects.

During the trial, 21 participants in the supplementation group (91.3%) showed an improvement in vitamin B1 status (from deficient to insufficient or adequate), and all participants showed an improvement in vitamin B2 status. In contrast, five participants (25%) in the placebo group showed improvement in vitamin B1 status, and six participants (30%) showed improvement in vitamin B2 status. This suggests that some of the participants in the placebo group may have taken other nutritional supplements outside of the period, whereas compliance was higher in the intervention group than in the control group. Since vitamin B1 and vitamin B2 levels are associated with changes in key outcome indicators (anxiety, stress, and sleep), cross-sectional studies are important.

### 3.3. Changes in Anxiety Scores Between the Vitamin B1 and B2 Supplementation Group and the Placebo Group

After 4 weeks of intervention (Table 4), there was no statistically significant difference in SAS scores between the supplementation group and the placebo group (*p* > 0.05). Within-group comparisons showed that the post-intervention anxiety score in the supplementation group was slightly lower than the baseline score (1.8, 95% CI: −1.7 to 5.4, *p* > 0.05), whereas the placebo group showed a slight increase in anxiety scores compared to baseline (−1.1, 95% CI: −7.1 to 5.1, *p* > 0.05). Although the supplementation group had marginally lower anxiety scores than the placebo group at week 4, the between-group difference did not reach statistical significance (adjusted *p* = 0.36) after adjusting for baseline SAS scores.

### 3.4. Vitamin B1 and B2 Supplementation Group Showed Improvement in Stress Perception Scores Compared to the Placebo Group

After 4 weeks of intervention, the PSS score was lower in the supplementation group (15.5 ± 4.5) than in the placebo group (19.8 ± 5.5). This between-group difference remained statistically significant after adjusting for baseline PSS scores (adjusted *p* < 0.005). Within-group analysis showed a significant reduction in stress perception scores in the supplementation group from baseline to week 4 (5.9, 95% CI: 4.4 to 7.4, *p* < 0.001), indicating the stress-reducing effect of the vitamin B1 and B2 supplementation. The placebo group showed a slight, non-significant reduction (0.5, 95% CI: −2.4 to 3.4, *p* > 0.05).

### 3.5. Improvement in Sleep Quality Index in Vitamin B1 and B2 Supplementation Group Compared with Placebo Group

After 4 weeks of intervention (Table 5), the PSQI score was lower in the supplementation group (6.3 ± 2.0) than in the placebo group (7.4 ± 2.9). This between-group difference remained statistically significant after adjusting for baseline PSQI scores (adjusted *p* = 0.018). Compared to the baseline, the PSQI index in the intervention group significantly decreased by 1.6 points (1.6, 95% CI: 0.49–2.9, *p* < 0.01), indicating improved sleep quality. In contrast, the placebo group showed a statistically significant increase in PSQI index compared to baseline (–1.6, 95% CI: −3.1 to −0.1, *p* < 0.05), suggesting worsened sleep quality.

### 3.6. Improvement in Daytime Sleepiness Scores in the Vitamin B1 and B2 Supplementation Group Compared to the Placebo Group

After 4 weeks of intervention, the ESS daytime sleepiness score was lower in the supplementation group (9.1 ± 3.9) than in the placebo group (10.3 ± 4.7). This between-group difference remained statistically significant after adjusting for baseline ESS scores (adjusted *p* = 0.001). Compared to baseline, the supplementation group showed a significant reduction in ESS scores (3.9, 95% CI: 2.8–5.0, *p* < 0.05), indicating improved sleepiness. In contrast, the placebo group showed a slight, non-significant decrease compared to baseline (0.3, 95% CI: −1.0 to 1.7, *p* = 0.61).

## 4. Discussion

### 4.1. Summary of Main Findings

This study examined the effects of vitamin B1 and Vitamin B2 nutritional supplements on reducing stress and anxiety and improving sleep quality in young people with vitamin B1 and vitamin B2 deficiencies. Our study’s findings demonstrated that taking a daily supplement of vitamin B1 and vitamin B2 for four weeks raised the nutritional levels of these two vitamins. The supplemented group showed significantly lower stress perception scores than the placebo group, suggesting that the supplemented group had a more substantial stress reduction. However, there was no statistically significant decrease in anxiety scores in the supplemented group following the intervention. A notable increase in sleep quality was indicated by the supplemented group’s significantly lower Sleep Quality Index when compared to the placebo group. Additionally, the supplement group’s post-intervention score showed a considerably lower daytime sleepiness score than the placebo group, suggesting that the supplement group also had a significant improvement in daytime sleepiness.

### 4.2. Examining the Findings in the Context of the Existing Literature

Our results are in line with other research on stress perception and sleep quality, which found an inverse relationship between high levels of stress, sleep risk, and vitamin B1 and vitamin B2 intake [28,29]. Similarly, a meta-analysis found that vitamin B1 and B2 help reduce stress and enhance the quality of sleep [47]. In support of this finding, a high dose of a multivitamin from the B family was found to significantly enhance stress levels after 33 days in a randomized, placebo-controlled, double-blind trial conducted by Kennedy et al. [48]. Furthermore, a randomized, placebo-controlled, double-blind trial by Zandifar et al. reported that vitamin B1 supplementation in patients with bidirectional affective disorder for 8 weeks resulted in a significant improvement in sleep quality compared to the placebo group [28]. Consistent findings have also been observed in cross-sectional studies. For example, vitamin B1 intake is higher in normal sleepers than in insomniacs and those with poor sleep quality [49]. Matsunaga et al. (2021) similarly observed a negative correlation between vitamin B1 intake and sleep quality index among young Japanese men [50]. Moreover, a study on the efficacy of vitamin B1 supplementation for acute COVID-19 syndrome showed that vitamin B1 supplementation for 5 weeks improved sleep disorders in patients [51]. These findings collectively reinforce our own results, particularly the significant improvements observed in sleep quality and stress perception following vitamin B1 and B2 supplementation (Table 3 and Table 4).

Our study’s findings demonstrated that there was no statistically significant difference in anxiety scores between and within the vitamin B1 and vitamin B2 supplementation and placebo groups, suggesting that there was no discernible impact of the supplements on anxiety reduction (Table 3). However, no statistically significant linear association was discovered between vitamin B1 and vitamin B2 and anxiety or depression in their large population-based cohort study [26]. Our results are in line with a randomized, placebo double-blind study that found no evidence of thiamine’s major impact on anxiety reduction [28]. However, vitamin B1 consumption was linked to a decreased risk of anxiety and depression in a Korean modeling study by Nguyen et al., which involved 9848 participants [52]. A large cross-sectional study of adolescent mental health in Western Australia showed that higher vitamin B1 and vitamin B2 intake was associated with lower scores on externalizing behaviors, reflecting the mitigating effects of vitamin B1 and vitamin B2 on anxiety [53]. This is not consistent with what this study found. This study noted that these variations might result from the use of various trial designs, dosages of vitamin B1 and vitamin B2 supplements, supplementation durations, and research populations. The current study found a slight decrease in anxiety scores in the supplemented group compared to the baseline period, but higher scores in the placebo group after the 4-week intervention than in the baseline period. But the differences in anxiety scores between the supplemented and placebo groups after the 4-week intervention were not statistically significant. The results indicated that the placebo group’s sleep quality became worse after four weeks. However, the placebo group had higher stress and sleepiness levels after four weeks, with no significant difference compared to baseline. This may have been influenced by lifestyle and social determinants, including academic performance and academic pressure. Previous studies have also shown that these factors are one of the determinants of sleep quality in college students [54,55].

According to Bouayed and Hassan et al., oxidative stress damage to the nervous system, impaired cellular energy metabolism, and impaired neurotransmitter metabolism are the causes of anxiety, stress, and poor sleep quality [56,57]. These factors can impair the nervous system’s ability to function normally, which can have an impact on mood and neurological health [56,57]. Some of the positive effects of vitamin B1 and vitamin B2 supplementation on mood states and sleep quality may be explained by the fact that these vitamins, whose mechanisms of action on mood and the brain are not fully understood, can protect the sensitive nervous system by participating in energy metabolism and preventing oxidative stress. There are four primary ways that thiamine deficiency (TD) is thought to work. The two primary dehydrogenase complexes that depend on thiamine diphosphate (TDP) are pyruvate dehydrogenase, which generates acetyl-coenzyme A and is involved in the aerobic metabolism of sugars, and the oxidative decarboxylation of α-ketoglutarate to succinyl-coenzyme A, which takes part in the tricarboxylic acid cycle. TDP is the active coenzyme form of thiamine. TDP is a precursor of thiamine, and TD lowers TDP levels and disrupts the action of essential enzymes, resulting in mitochondrial malfunction, which is crucially connected to cerebral activity and the cellular energy supply [58,59]. In addition, vitamin B1 plays a role in the production of several neurotransmitters, including acetylcholine, γ-aminobutyric acid, glutamate, aspartate, and serotonin. TD aggravates neurotransmitter synthesis imbalances, which can affect mood and intensify stress and anxiety reactions [34]. TD also aggravates oxidative stress damage, which causes neurological damage and a decrease in hippocampus volume because of the nervous system’s heightened sensitivity to oxidative stress [17]. Since vitamin B1 is a necessary cofactor for the metabolism of glucose, TD may affect the brain’s poor energy metabolism, cause neuronal damage, and trigger an inflammatory response that makes people more prone to mood swings, weariness, and anxiety [60]. Anxiety and stress brought on by TD make sleep disruptions worse, and since little sleep feeds anxiety and stress, the cycle is vicious. This implies that vitamin B1 may have an impact on neurological health and mood stabilization.

Furthermore, vitamin B1 and vitamin B2 work in tandem to support energy metabolism and neuroprotection. Two coenzymes that are essential for cellular energy metabolism and ATP synthesis are FMN (flavin mononucleotide) and FAD (flavin adenine dinucleotide), which can be produced from riboflavin [61]. Given how sensitive the brain is to energy demands, FMN and FAD can act as electron acceptors or donors in redox reactions within the mitochondrial electron transport chain, facilitating the production of ATP, supplying enough energy for neurons, and supporting the synthesis and release of neurotransmitters—all of which are essential for reducing stress and anxiety [62]. FAD functions as a cofactor for succinate dehydrogenase, which participates in the succinate oxidation pathway in the tricarboxylic acid cycle. By accepting electrons, FAD creates flavin adenine dinucleotide reduced form (FADH_2_), which facilitates the electron transport chain’s ATP generation. Glutathione reductase (GSSG reductase) converts oxidized glutathione (GSSG) to reduced glutathione (GSH) with the help of vitamin B2 as a cofactor. GSH, an important antioxidant in the body, acts on free radicals to reduce oxidative stress and protect cells from free radical damage [63]. Therefore, vitamin B2 may aid in lowering mood problems linked to stress, anxiety, and, indirectly, the quality of sleep.

These results imply that taking vitamin B1 and vitamin B2 supplements could be a useful tactic to reduce stress and improve sleep. Vitamin B1 and vitamin B2 are more widely accepted as natural vitamins with safe, natural, and non-pharmacological treatments than as therapeutic medications [39,64]. A randomized, placebo-controlled trial by Dean et al. involving a 16-week combination therapy (including 100 mg/day of thiamine and riboflavin) for depressive episodes in bipolar disorder found that the study results were considered to offer new possibilities for adjunctive therapy [37]. Dean et al. noted that since nutritional supplements are typically sold as over-the-counter products and do not require additional regulatory approval, study participants could continue taking the supplement (or begin taking it in the placebo group) after the trial concluded [37]. This not only benefits participants’ health but also highlights the potential of nutritional supplements in real-world clinical applications. Additionally, vitamin B1 and vitamin B2 can be found in a wide range of natural foods, such as whole grains, legumes, and animal liver, in addition to taking supplements [24,65]. By changing dietary habits and cooking techniques (more boiling, steaming, and stir-frying), the need for vitamin B1 and vitamin B2 supplements can be satisfied. This implies that vitamin B1 and vitamin B2 can be supplemented in several methods that are appropriate for the lifestyles of various groups, in addition to being helpful for those who suffer from stress, poor sleep quality, and severe sleepiness.

### 4.3. Limitations and Future Research Directions

Due to the relatively small sample size, the statistical results may not be fully generalizable and may not have sufficient statistical power to detect subtle effects, thereby increasing the risk of type II errors. Although this study performed an analysis of covariance for the primary outcome variables, no correction for multiple comparisons (e.g., Bonferroni) was performed, which may have increased the risk of false positives. Secondly, due to research constraints and ethical considerations, this study did not measure key biomarkers such as serotonin, cortisol, C-reactive protein, or other relevant indicators in participants, thereby limiting the ability to draw conclusions regarding underlying mechanisms. Nonetheless, these limitations open avenues for future research to explore these factors and their roles in the observed outcomes.

Since the supplementation lasted only four weeks, it may not have had a sufficient impact on the measurement of issues. Vitamins B1 and B2 may require longer to begin to influence anxiety. As the duration of the intervention, this study may challenge the assessment of long-term effects. One limitation of this study is that the sample was exclusively composed of individuals with relatively high levels of anxiety, stress, and sleep index. This selective inclusion may limit the generalizability of the results to the broader population. This research did not consider genetic differences between individuals, and genetic factors may play a role in the effectiveness of interventions. Future studies should consider the potential influence of genetic factors on the response to nutritional supplementation interventions. We did not measure dietary factors and physical activity, which could introduce variability in the outcomes.

Although B vitamins are water-soluble and participants were instructed to take them at the same time, we acknowledge that consuming them with certain foods may affect their bioavailability. While participants in this study all came from a population with an Eastern dietary background, it is important to recognize that variations in individual dietary habits may still have influenced the results. This illustrates how larger sample sizes, populations with different dietary patterns, longer intervention and follow-up periods, varying intervention doses, and more thorough mechanistic investigations—such as additional validation of inflammatory factors, neurotransmitters, and oxidative stress levels—may enable future research to broaden the study’s coverage.

## 5. Conclusions

In conclusion, the study’s results demonstrate that taking vitamin B1 and B2 supplements can successfully reduce stress, enhance sleep, and ease sleepiness, but they have no significant effect on reducing anxiety. Based on the data presented in this study, taking vitamin B1 and B2 supplements may be an effective approach for reducing stress and enhancing sleep, providing a safe, natural, and affordable way to deal with the major health burden facing modern society. Future research could further evaluate the effects of vitamin B1 and B2 supplements in long-term interventions, particularly in combination with medication, on the comprehensive improvement of stress-related disorders and sleep disorders. Developing precision nutrition intervention strategies for vitamins B1 and B2 based on individual physiological characteristics and metabolic differences will help enhance the targeting and effectiveness of interventions.

## Figures and Tables

**Table 1:** Participant characteristics at baseline.

Variables	Vitamin B1 and B2 Group	Placebo Group
Sociodemographic		
N	23	20
Age, y	19.5 ± 1.2	19.7 ± 1.5
Sex, n (%)		
Female	12 (52.2%)	12 (60%)
Male	11 (47.8%)	8 (40%)
Native place, n (%)		
Northern China	5 (21.8%)	2 (10%)
South China	18 (78.2%)	18 (90%)
≤College junior education, n (%)	12 (52.2%)	10 (50%)
Lifestyle		
BMI, kg/m^2^	22.9 ± 2.5	21.4 ± 3.2
Daily physical activity practician (%)		
Mild level	21 (91.3%)	17 (85%)
Middle level	1 (4.3%)	2 (10%)
Severe level	1 (4.3%)	1 (5%)
Smoking, yes (%)	3 (13%)	0 (0%)
Drinking, yes (%)	2 (8.6%)	1 (5%)
Nutritional status		
Vitamin B1 in urine,ug	158.8 ± 108.9	113 ± 68.9
Vitamin B2 in urine,ug	308 ± 198.3	309.4 ± 190.4
Dietary consumption		
Dietary consumption per day, yuan	130 ± 39.9	108 ± 33
Consumption per meal, yuan		
Breakfast	6.6 ± 8.8	7.9 ± 10
Lunch	37.3 ± 12.6	39.3 ± 11.4
Dinner	38.7 ± 15.5	42 ± 15.1
Midnight snack	5.4 ± 11.4	4.4 ± 8.6
Drink and snack	18.2 ± 12.6	17.4 ± 10.6

Data are presented as mean ± standard deviation (SD) for continuous variables and as frequency (percentage) for categorical variables. BMI, body mass index.

**Table 2:** Between-group and within-group comparison changes in urine levels of Vitamin B1 of study participants.

	Vitamin B1 and B2 Group	Placebo Group	*p*-Value ^a^	Adjusted *p*-Value ^d^
Baseline (ng)	158.8 ± 108.9	167.6 ± 125.0	0.609	0.039 ^c^
After 4 weeks	1333.1 ± 1204.5	408.3 ± 299.7		
Mean difference (95% CI)	−808.0 (−1075.7, −540.3)	−240.5 (−461.2, −19.8)		
*p*-value ^b^	<0.001 ^c^	0.036 ^c^		

Data are shown as mean ± SD. ^a^ Obtained from an independent sample *t*-test based on comparing the mean differences between two groups at baseline. ^b^ Obtained from a paired sample *t*-test based on comparing the mean differences between two different conditions for the same set of samples. ^c^ Significant statistical difference (*p* < 0.05). ^d^ Obtained from ANCOVA comparing post-intervention vitamin B1 levels between groups, adjusted for baseline BMI, age, and smoking status.

**Table 3:** Between-group and within-group comparison of changes in urine levels of vitamin B2 of study participants.

	Vitamin B1 and B2 Group	Placebo Group	*p*-Value ^a^	Adjusted *p*-Value ^d^
Baseline (ng)	308.0 ± 198.3	309.4 ± 109.4	0.980	0.004 ^c^
After 4 weeks	6123.2 ± 4847.2	372.0 ± 209.7		
Mean difference(95% CI)	−5831.8 (−8283.4, −3380.6)	−39.0 (−167.1, 83.6)		
*p*-value ^b^	<0.001 ^c^	0.509		

Data are shown as mean ± SD. ^a^ Obtained from an independent sample *t*-test based on comparing the mean differences between two groups at baseline. ^b^ Obtained from a paired sample *t*-test based on comparing the mean differences between two different conditions for the same set of samples. ^c^ Significant statistical difference (*p* < 0.05). ^d^ Obtained from ANCOVA comparing post-intervention vitamin B2 levels between groups, adjusted for baseline BMI, age, and smoking status.

**Table 4:** Within-group and between-group comparison of changes in the mean score of the SAS, PSS.

		Vitamin B1 and B2 Group	Placebo Group	*p*-Value ^a^	Adjusted *p*-Value ^d^
Mean score of SAS	Baseline	45.8 ± 10.2	44.1 ± 11.6	0.67	0.36
	After 4 weeks	44.0 ± 6.0	45.1 ± 13.2		
	Mean difference (95% CI)	1.8 (−1.7, 5.4)	−1.1 (−7.1, 5.1)		
	*p*-value ^b^	0.294	0.730		
Mean score of PSS	Baseline	21.5 ± 4.1	20.3 ± 4.3	0.37	<0.005 ^c^
	After 4 weeks	15.5 ± 4.5	19.8 ± 5.5		
	Mean difference (95% CI)	5.9 (4.4, 7.4)	0.5 (−2.4, 3.4)		
	*p*-value ^b^	<0.001 ^c^	0.725		

Data are shown as mean ± SD. ^a^ Obtained from an independent sample *t*-test based on comparing the mean differences between two groups at baseline. ^b^ Obtained from a paired sample *t*-test based on comparing the mean differences between two different conditions for the same set of samples. ^c^ Significant statistical difference (*p* < 0.05). ^d^ Obtained from ANCOVA comparing post-intervention values between groups, adjusted separately for baseline SAS or PSS scores.

**Table 5:** Within-group and between-group comparison of changes in the mean score of the PSQI, ESS.

		Vitamin B1 and B2 Group	Placebo Group	*p*-Value ^a^	Adjusted *p*-Value ^d^
Mean score of PSQI	Baseline	8.0 ± 3.2	5.7 ± 2.7	0.019 ^c^	0.018 ^c^
	After 4 weeks	6.3 ± 2.0	7.4 ± 2.9		
	Mean difference(95% CI)	1.6 (0.49, 2.9)	−1.6 (−3.1, −0.1)		
	*p*-value ^b^	0.008 ^c^	0.037 ^c^		
Mean score of ESS	Baseline	13.0 ± 3.4	10.6 ± 4.2	0.04 ^c^	0.001 ^c^
	After 4 weeks	9.1 ± 3.9	10.3 ± 4.7		
	Mean difference(95% CI)	3.9 (2.8, 5)	0.3 (−1, 1.7)		
	*p*-value ^b^	<0.001 ^c^	0.61		

Data are shown as mean ± SD. ^a^ Obtained from an independent sample *t*-test based on comparing the mean differences between two groups at baseline. ^b^ Obtained from a paired sample *t*-test based on comparing the mean differences between two different conditions for the same set of samples. ^c^ Significant statistical difference (*p* < 0.05). ^d^ Obtained from ANCOVA comparing post-intervention values between groups, adjusted separately for baseline PSQI or ESS scores.

## Data Availability

The datasets generated and/or analyzed during the current study are not publicly available to protect participant privacy but are available from the corresponding author on reasonable request.
